# A Dual Role for the Placenta and Human Amniotic Epithelial Cells in HER3-Targeted Nanotherapy

**DOI:** 10.21203/rs.3.rs-9107787/v1

**Published:** 2026-05-20

**Authors:** Amirhesam Babajani, Kristin Ishaya, Joseph Aceves, Nelyda Gonzalez-Almeyda, Abby Wiesenthal, Ravinder Abrol, Lali Medina-Kauwe

**Affiliations:** Cedars-Sinai Medical Center; Cedars-Sinai Medical Center; Cedars-Sinai Medical Center; Cedars-Sinai Medical Center; Cedars-Sinai Medical Center; CSUN: California State University Northridge; Cedars-Sinai Medical Center

**Keywords:** ErbB-3, Amnion, Placenta, Umbilical Cord, Epithelial Cells, Extracellular Vesicles, Drug Delivery Systems, Maternal-Fetal Exchange

## Abstract

**Background:**

HER3-targeted therapies are developing to clinical trials, while late pregnancy and earlier-onset breast neoplasm have increased the chance of overlap between childbearing age and cancer incidence. While more cancer patients of childbearing age may be exposed to these agents, considering HER3 expression and interactions within placental tissues is crucial for maternal and fetal safety.

**Methods:**

Human placentas from healthy deliveries were used to isolate human amniotic epithelial cells (hAECs) and prepare amniotic membrane and umbilical cord sections. HER3 expression was analyzed in tissues, cells, and exosomes by multiplex immunofluorescence, flow cytometry, and immunoblot. The HER3-targeted nano-capsomere HPK2.0 and its nucleic acid-complexed form, HerLLAA, were synthesized and assessed by dynamic light scattering, and impact on hAEC viability was evaluated by metabolic assay. HPK uptake, release, and intracellular trafficking were examined using pulse-chase quantification and time-resolved imaging.

**Results:**

Freshly isolated hAECs exhibited epithelial morphology and heterogeneous stemness and mesenchymal marker expression, with a substantial HER3-positive population. HER3 was strongly localized to the amniotic membrane epithelial layer and was enriched in the umbilical cord endothelium compared to vessel wall and Wharton’s jelly. hAEC-derived extracellular vesicles displayed stable size profiles and contained HER3. HPK2.0 and HerLLAA formed stable nano-structures without inducing cytotoxicity on hAECs. Functionally, hAECs rapidly internalized HPK2.0 and HerLLAA and released it in a dose- and time-dependent manner, consistent with regulated intracellular trafficking rather than long-term retention.

**Conclusions:**

The amniotic membrane, umbilical cord, hAECs, and their exosomes express HER3 and actively interact with HER3-targeted nanotherapeutics. HPK2.0 and HerLLAA undergoes regulated uptake and release without cytotoxicity, underscoring both safety considerations during pregnancy and the potential for HER3-mediated placental and fetal drug delivery.

## Introduction

Human Epidermal Growth Factor Receptor 3 (HER3 or ERBB3) is a member of the ERBB receptor tyrosine kinase family, which also includes EGFR (HER1), HER2, and HER4. Unlike its family members, HER3 has an impaired intracellular kinase domain, and thus little to no intrinsic catalytic activity^1^. However, it plays a critical role in cancer biology by forming heterodimers– particularly with HER2 or EGFR– that activate key signaling pathways such as PI3K/AKT and MAPK, driving tumor cell survival, proliferation, and therapeutic resistance^2,3^. Recent research has emphasized HER3 as an important mechanism of resistance to existing HER2- and EGFR-targeted therapies in a vast majority of cancers, including breast, lung, and colorectal malignancies^4–6^.

The emergence of HER3-targeting agents has introduced a promising therapeutic strategy in oncology. Advances in understanding the role of HER3 in cancer have led to the development of targeted modalities -- including monoclonal antibodies^7,8^, antibody-drug conjugates (ADCs)^9,10^, bispecific antibodies^10^, nanoparticles^11,12^, and vaccines^13^ – designed to disrupt HER3 signaling or exploit its selective expression on tumor cells. Among these, Patritumab deruxtecan (HER3-DXd) has demonstrated compelling activity in EGFR-mutant non-small cell lung cancer and other solid tumors, highlighting the clinical potential of HER3 as a therapeutic target^14^. Similarly, bispecific antibodies such as Zenocutuzumab, which targets both HER2 and HER3, have shown considerable responses in NRG1 fusion-positive tumors, a genomically defined patient subset^15^. In addition, our novel recombinant chimeric protein, HerPBK10 (HPK), a HER3-targeted nano-capsomere engineered to bind and encapsulate anionic therapeutic cargo, enables efficient and selective delivery to resistant and metastatic HER3^+^ cancer cells^16,17^.

Most HER3-targeting therapies are still in preclinical or early clinical development; therefore, their use in clinical practice should be approached with caution due to potential side effects. A key population requiring particular attention when considering new therapies includes women of childbearing age and pregnant women^18^. Pregnancy-related cancers occur predominantly in the breast, and their incidence continues to rise as delayed childbearing increases both risk and adverse outcomes in younger patients. With the progressive rise in women’s average childbearing age, the age distributions for pregnancy and breast cancer diagnosis now significantly overlap^19^. HER3-targeting agents may offer therapeutic options for women of childbearing age and pregnant women, particularly for resistant cancers including breast cancer; however, their use requires further evaluation due to the unique physiological conditions of pregnancy and the critical importance of fetal safety.

The placenta is the primary organ that connects the maternal and fetal circulatory systems. It has evolved to support the growth and development of the embryo and fetus throughout the different stages of intrauterine life. The placenta is composed of several distinct parts that work together to fulfill this vital function^20^. One important component is the amniotic membrane (AM), which plays a key role in protecting and supporting the fetus. AM is composed of two distinct tissue layers: (1) an inner single-layered amniotic epithelium that lines the uterine cavity that remains in direct contact with the amniotic fluid and fetus; and (2) an outer chorionic mesenchymal layer that interfaces with the maternal decidua, forming the feto-maternal barrier^21^. Human amniotic epithelial cells (hAECs) from the innermost layer serve as a physical barrier, protecting the fetus from pathogens, toxins, and mechanical stress. They also secrete a diverse array of cytokines, growth factors, and extracellular vesicles (EVs)^22^. Experimental studies suggest that amniotic fluid is primarily regulated by the amniotic layer, through the rapid transfer of water and solutes across the placental amnion into the fetal circulation. In hAECs, vesicular uptake of macromolecules occurs rapidly, indicating that vesicular transcytosis across the amnion contributes to the regulation of amniotic fluid volume and content^23^. In support, hAECs exhibit the ability to internalize chemotherapeutic agents and subsequently secrete them in a time dependent manner, indicating that these cells implement a regulated vesicular handling of exogenous compounds^24^.

Considering these properties, hAECs represent a dynamic cellular system that actively engages with therapeutic macromolecules rather than serving as a passive barrier^23^. Importantly, this raises the possibility that HER3-targeted nanocarriers such as HPK, which bind anionic cargos and undergo receptor-mediated internalization^11,16^, may traverse or be processed by hAECs in a controlled manner. Such interactions could influence both placental exposure and the potential delivery of therapeutic cargos to placental or fetal compartments. Understanding how HPK interacts with hAECs is therefore critical not only from a safety perspective, but also for evaluating whether HER3-targeted nanocarriers could be adapted for controlled therapeutic delivery to the placenta. In this study, we evaluate HER3 expression in the placenta tissues and on hAECs and their secreted EVs. We also evaluate the capacity of these cells to internalize the HPK nanocarrier, and assess their ability to subsequently release it into the surrounding environment. Additionally, we track the intracellular dynamics of HPK within hAECs to assess potential implications for placental drug transport and fetal exposure.

## Materials and Methods

### Placenta Tissue Processing

The process of hAECs isolation was performed based on previous studies^24,25^. After healthy mothers delivered and provided informed consent, human placentae were received via the Cedars-Sinai Biobank as unidentified samples, moved to the laboratory under aseptic conditions, and maintained at controlled temperature. All experiments were carried out following approval from the Office of Research Compliance and Quality Improvement, Cedars-Sinai Medical Center (Approval ID: Pro00048705), adhering to all relevant guidelines and regulatory standards.

Following mechanical separation from the chorion, the amniotic layer was rinsed with cold PBS to remove residual blood and debris. The AM was then dissected into small pieces and incubated with 0.15% trypsin-EDTA (Gibco, Canada) at 37°C for 10 min. Cells from this first digestion were discarded to remove debris. Supernatants were collected from the second and third 40-minute digestions, and trypsin activity was inactivated with FBS (Gibco, Canada). The resulting cell suspension was centrifuged at 500 × g for 5 min, and hAECs were resuspended in Dulbecco’s Modified Eagle Medium (Gibco, Canada) supplemented with 10% FBS, 100 U/mL penicillin/streptomycin (Gibco, Canada), and 10 ng/mL human epidermal growth factor (hEGF) (Sigma-Aldrich, Israel).

### Flow Cytometry

hAECs were cultured in complete media and used at 70–80% confluence. Following harvesting and PBS washes, cells were fixed for 15 minutes in 4% (w/v) paraformaldehyde prior to immunolabeling. Antigen expression was analyzed using primary antibodies against stemness markers stage-specific embryonic antigen-4 (SSEA-4) (clone MC-813–70, Thermo Fisher Scientific, RRID: AB_2533506) and OCT4 (clone 9B7, Thermo Fisher Scientific, catalog # MA1–104, RRID: AB_2536771). In addition, mesenchymal markers CD105 (clone 3A9, Thermo Fisher Scientific, catalog # MA5–17041, RRID: AB_11155625), CD90 (clone F15-42-1, Thermo Fisher Scientific, catalog # MA5–16671, RRID: AB_2538810), and CD73 (clone 7G2, Thermo Fisher Scientific, catalog # 41–0200, RRID: AB_2533397), as well as HER3/ErbB3 (polyclonal, Thermo Fisher Scientific, catalog # PA5–14636, RRID: AB_2099571), were evaluated. For intracellular antigen detection, cells were permeabilized after fixation for 15 min using 0.1% Triton X-100 and 3% (w/v) BSA in PBS, followed by blocking for 1 h with 3% (w/v) BSA in PBS and antibody staining. Primary antibodies were diluted using 3% (w/v) BSA in PBS, incubated overnight, and detected using Alexa Fluor-conjugated secondary antibodies. Flow cytometry analysis was performed using a Moxi Flow cytometer with standardized voltage and compensation settings. Unstained and secondary-only controls were included to define baseline fluorescence and gating thresholds.

### Exosome Isolation

hAECs were cultured in complete media until reaching 70–85% confluency. The culture medium was aspirated. Subsequently, the cells were rinsed with plain medium to remove residual vesicles. After that, 12–16 mL of exosome-depleted medium was added to each T150 flask, and the cells were incubated for 24 h. The conditioned medium (CM) was then collected and transferred to 50-mL tubes. CM from multiple flasks were pooled when necessary and was sequentially centrifuged 300 × g for 5 min to remove cells and 3,500 × g for 15 min to remove debris. The clarified supernatant was filtered through a 0.20-μm syringe filter (Cytivia, USA) to remove remaining particulates and stored at 4°C for up to 3 days before further processing. To concentrate samples, 100 kDa centrifugal ultrafiltration devices (Amicon Ultra, Ireland) were pre-soaked overnight in 10% glycerol to reduce non-specific vesicle binding. The clarified supernatant was loaded into the ultrafiltration devices and centrifuged at 3,500 × g for 20 min. The process was repeated until the entire volume of supernatant was concentrated (100–150 μL). The concentrated CM was then mixed with the Total Exosome Isolation Reagent from cell culture media (Thermo Fisher, USA) at a ratio of 0.5 volumes of reagent per 1 volume of concentrated CM. The mixture was homogenized by gentle vortexing and incubated overnight at 4°C. After incubation, samples were centrifuged at 10,000 × g for 1 hour at 4°C. The supernatant was carefully removed, and the resulting exosome pellet was resuspended in 100 μL of 0.20 μm-filtered PBS. The isolated exosomes were stored at −80°C for long-term preservation.

### Western Blot

To evaluate ErbB3/HER3 levels in hAECs-derived exosomes, total protein concentrations of exosome samples from week 1 to week 3 were verified using the Pierce^™^ BCA assay. Equal amounts of 20 μg protein were then injected into SDS-PAGE followed by western blotting to assess ErbB3/HER3 expression. Protein samples were combined with 4X Laemmli buffer, denatured at 95°C for 7 minutes, briefly centrifuged, and kept on ice prior to loading. Precision Plus Protein Unstained and All Blue standards (1:1) served as molecular weight markers. Proteins were separated on precast gels using a Mini-PROTEAN system in 1X SDS running buffer and visualized under stain-free conditions on a ChemiDoc MP system before transfer.

Proteins were transferred to nitrocellulose membranes via wet transfer at 25 V for 30 minutes using 1X Transfer Buffer, prepared with 20% Trans-Blot Turbo 5X Transfer Buffer (Bio-Rad, catalog #10026938) and 20% ethanol in water. Membranes were blocked for 1 hour with 3% (w/v) BSA in PBS and subsequently incubated overnight at 4°C with primary antibodies diluted 1:1,000 in 3% (w/v) BSA in PBS. The primary antibodies used were polyclonal anti-ErbB3/HER3 (Thermo Fisher Scientific, catalog # PA5–14636, RRID: AB_2099571), polyclonal anti-CD63 (Sigma-Aldrich, catalog # HPA010088). “After washing with TBS-T and TBS, membranes were incubated for 1 hour with HRP-conjugated polyclonal secondary antibodies (Goat Anti-Rabbit IgG H&L, Cat. No. ab6721, Abcam) diluted 1:5,000 in 3% (w/v) BSA in PBS. After additional washes, signals were developed using Clarity Western ECL Substrate (Bio-Rad, catalog # 170–5061). Chemiluminescent signals were detected with a ChemiDoc MP system under high-sensitivity settings. The images were analyzed using ImageJ software.

### Nanoparticle Tracking Analysis (NTA)

To characterize the isolated exosomes, their size distribution and concentration were measured using NTA on a NanoSight instrument (Malvern Panalytical, UK). Before analysis, exosome samples were thawed on ice and centrifuged at 3,500 × g for 10 min at 4°C to remove aggregates. The clarified supernatant was diluted in 0.20 μm-filtered PBS to obtain an appropriate working concentration. Prior to sample measurement, the instrument was flushed with particle-free PBS, and a baseline background reading was verified. For each sample, three 60 s videos were recorded under constant flow using a syringe pump. Camera level (12–15) and detection threshold (5–10) were kept constant for all measurements. Analyses were performed at room temperature (25°C). Raw particle trajectories were processed using NTA software (version 3.4). Data are reported as mean particle size ± SD and particle concentration (particles/mL).

### Immunofluorescence Staining

After receiving eligible placentas, samples of tissue were harvested from the maternal and fetal sides of the umbilical cord (UC) and from the AM, and followed by fixation in 4% (w/v) paraformaldehyde for 48–72 h. Paraffin-embedded tissue blocks (FFPE) were prepared from the fixed tissues by the Biobank. The FFPE blocks were sectioned using a microtome, and the slides were dried at room temperature for 48 h. FFPE sections were deparaffinized in Xylene, rehydrated through a graded ethanol series, and fixed with 10% Neutral Buffered Formalin for 20 min. Heat-induced antigen retrieval was performed at 95°C using 1X Tris-EDTA buffer (pH 9.0) with 0.05% (v/v) TWEEN-20 for the first retrieval round and 1X Citrate buffer (pH 6.0) with 0.05% (v/v) TWEEN-20 for subsequent rounds. After cooling and washing in 1X PBS, tissue sections were blocked with 5% normal animal serum with 1% (w/v) BSA in PBS-T for 1 h at room temperature and incubated with primary antibodies against pan-cytokeratin (pan-CK; clone AE1 + AE3, Thermo Fisher Scientific, catalog # CF190321) and ErbB3/HER3 (polyclonal, Thermo Fisher Scientific, catalog # PA5–14636). Antibodies were diluted in PBS containing 3% (w/v) BSA with 0.1% TWEEN-20 in PBS (1:100) and subsequently incubated overnight in a moisture-controlled chamber at 4°C. Following PBS-T washes, Alexa Fluorophore-conjugated secondary antibodies were diluted in 3% BSA with 0.1% TWEEN-20 in PBS (1:500) and placed on the slides for 30 minutes under ambient conditions for pan-CK (AF-488 Goat anti-Mouse, Thermo Fisher Scientific, catalog # A-11001) and for HER3 (AF-680 Donkey anti-Rabbit, Thermo Fisher Scientific, catalog # A-10043) detection. Tissue autofluorescence was quenched using 1X TrueBlack Lipofuscin Autofluorescence Quencher in 70% ethanol for 30 s and washed with 1X PBS. After the first round of antigen-retrieval only, tissues were fixed with 4% (w/v) Paraformaldehyde in PBS for 10 min and subsequently washed with 1X PBS. After all rounds of staining were complete, the slides were mounted with ProLong Gold Antifade Reagent with DAPI prior to imaging. Images were acquired using an ImageXpress Pico scanner (Molecular Devices) to assess pan-CK and HER3 levels. Image acquisition and assembly were performed using MetaXpress software, and image analysis was conducted using ImageJ.

### HPK2.0 Production

Recombinant proteins were produced based on previous studies using the pRSETA bacterial expression vector, which introduces an N-terminal 6×His tag for metal chelate affinity purification^11,16^. *Escherichia coli BLR(DE3)pLysS* cells underwent transformation with pRSET constructs were initially cultured in 50-mL LB medium at 33°C with vigorous shaking for 16 h and then expanded to 500-mL cultures. Protein expression was induced with 0.4 mM IPTG at an optical density (OD_600_) of 0.6–0.8 and allowed to proceed for 3 h. Following centrifugation, cells were resuspended in lysis buffer (50 mM NaH_2_PO_4_, 50 mM NaCl, pH 8.0) containing 0.1% Triton X-100 and 1 mM PMSF. Lysates underwent a single freeze-thaw cycle, after which MgCl_2_ (10 mM) and DNase I (0.01 mg/mL) were introduced and gently agitated at room temperature until viscosity was reduced. Lysates were placed on ice, adjusted to final concentrations of 1 M NaCl and 10 mM imidazole, and clarified by centrifugation at 39,000 × g at 4°C. His-tagged proteins were purified from cleared lysates utilizing nickel-charged affinity media and eluted with a stepwise imidazole gradient (20–250 mM) in 50 mM NaH_2_PO_4_ and 300 mM NaCl. Fractions containing the target protein (~ 92 kDa) were pooled and buffer-exchanged by ultrafiltration into storage buffer (20 mM HEPES, pH 7.4, 150 mM NaCl, 10% glycerol). Purified proteins were qualified by dynamic light scattering (DLS) and stored at −80°C until use.

### HerLLAA Production

We prepared HerLLAA complexes by using purified HPK2.0 protein and LLAA oligonucleotide. Components were thawed on ice and combined at a 6:1 molar ratio (HPK2.0:LLAA), based on molecular weights of 78 kDa and 10 kDa, respectively. Mixtures were incubated and mixed overnight at 4°C to allow complex formation, followed by filtering by 50 kDa molecular weight centrifugal filters. Final concentrations were measured by NanoDrop, and complex formation and particle integrity were confirmed by dynamic light scattering (DLS)

## DLS

DLS measurements were performed using a Malvern ZEN 3600 Zetasizer Nano. For each sample, a minimum of seven independent measurements was acquired, with each measurement consisting of 100 runs and an average particle count rate of approximately 34,000 counts per second. Particle size was reported as the number-weighted mean diameter, representing the most frequently occurring particle population while minimizing bias from high-intensity scattering by larger particles. Data analysis was conducted using Zetasizer software version 7.01, which calculates particle size by applying the Stokes-Einstein equation to relate fluctuations in scattering intensity to Brownian motion.

### HPK2.0 Metabolic Assay

HPK-induced cytotoxicity was assessed using an MTS assay. A total of 10,000 hAECs per well were plated in 96-well plates with 100 μL of complete medium and allowed to adhere overnight. Cells were treated for 24 h with HerLLAA at 300 − 1.17 μg/mL prepared by two-fold serial dilution. After adding 10 μL of MTS Assay Kit (Cell Proliferation Colorimetric, Cat. No. ab197010) reagent to each well, samples were incubated at 37°C for 3 hours, and absorbance was recorded at 490 nm.

### HPK2.0 Uptake and Release Assay

hAECs were seeded in 24-well plates at a density of 7–10 × 10^4^ cells per well in 500 μL complete medium and incubated overnight at 37°C in 5% CO_2_. For the uptake (pulse) phase, cells were washed once with 500 μL warm PBS and incubated with HPK diluted in basal medium containing 1% exosome-depleted FBS. A two-fold serial dilution of HPK was prepared to generate final treatment concentrations ranging from 80 to 5 μg/mL. Cells received 500 μL of each HPK dilution and were incubated for 1 h at 37°C. Control wells included hAECs treated with pulse medium lacking HPK and HPK-containing pulse medium incubated in empty wells to assess HPK stability and background binding. Following the 1 h pulse, the conditioned medium was collected and transferred to labeled microtubes, and cells were washed three times with 500 μL warm PBS to remove unbound HPK. The uptake phase was immediately followed by the release phase, in which 500 μL of complete medium containing 10% exosome-depleted FBS (HPK-free) was added to each well. Conditioned media were collected at 0.5, 1, 6, and 24 h after the chase began. At each timepoint, the medium was gently mixed clarified by centrifugation at 300× g for 5 min to remove cells. Supernatants were stored at − 20°C until analysis.

For quantification of intracellular HPK uptake at the final timepoint (24 h), cells were washed once with PBS and lysed in 100 μL lysis buffer (1% Triton X-100, supplemented with protease inhibitor). Lysates were incubated on ice for 10 min, scraped, collected, and clarified by centrifugation at 20,000 × g for 20 min. Supernatants were stored at − 20°C for HPK quantification assay.

### Quantification of HPK2.0

Quantification of His-tagged HPK2.0 in cell lysates and conditioned media was performed using Ni-NTA-coated 96-well plates (Cat. No. 15142; Thermo Fisher Scientific) according to the manufacturer’s instructions. All reagents were prepared in PBS (Dilution Buffer), and plate washes were performed with PBS containing 0.05% Tween-20 (Wash Buffer). Because the plates are pre-blocked with BSA, no additional blocking steps were performed. Frozen pulse and chase supernatants and cell lysates were thawed on ice. An HPK standard curve (0–200 ng/mL) was prepared in PBS. The Ni-coated plate was equilibrated to room temperature, and 100 μL of either standards or samples was added to each well. Plates were incubated for 1 h at room temperature with gentle shaking to allow His-tagged HPK to bind the immobilized Ni^2+^. Wells were then washed three times with 200 μL Wash Buffer.

To detect bound HPK, wells were incubated with 100 μL of anti-AD5 primary antibody (Cat. No. ab6982; Abcam), diluted 1:1,000 in PBS, for 1 hour at room temperature under gentle agitation, followed by three washes with Wash Buffer. Subsequently, 100 μL of HRP-conjugated secondary antibody diluted in PBS was added for 1 h under the same conditions, followed by three additional washes. Signal was developed by adding 100 μL of TMB substrate. Reactions were stopped with acidic stop solution and absorbance was measured at 450 nm. HPK concentrations in samples were interpolated from the standard curve.

Uptake percentage was defined as the proportion of HPK internalized by hAECs relative to the total HPK initially applied during the pulse phase. Cumulative release was calculated as the sum of HPK released into the conditioned medium at all measured time points during the chase period (0.5–24 h). Reduction release represented the remaining HPK pool after release and was calculated as the initial intracellular HPK amount minus the cumulative HPK released up to each time point Release percentage was defined as the fraction of HPK released at a given time point or cumulatively, normalized to the initial intracellular HPK amount.

### Cell Trafficking

HPK binding and intracellular trafficking were determined using a chamber slide *in vitro* assay. Four wells of a 4-well chamber slide were seeded with 15,000 hAECs per well and allowed to adhere and grow in the wells for 24 h. For the binding phase, the culture medium was discarded, and a single wash with ice-cold PBS was performed. The cells were incubated on ice in 250 μL of Buffer A containing 0.15 nmol of HPK in a final volume of 250 μL per well. Buffer A consisted of DMEM lacking serum and antibiotics, 20 mM HEPES (pH 7.4), 2 mM MgCl_2_, and 3% BSA. After the incubation of the cells on ice for 1 h, the wells for the 0 min time point were processed for fixation, while the other wells were removed from the ice and placed in a 37°C incubator in pre-warmed complete DMEM. At each point, the slides were washed three times with PBS containing 1% MgCl2 for 5 minutes. The cells then were fixed with 4% paraformaldehyde solution at pH 7.4 and incubated for 15 minutes at room temperature. The cells then were washed with PBS and incubated with 50 mM ammonium chloride solution for 5 minutes to quench autofluorescence. The slides then were washed and incubated with PBS containing 1% BSA for 1 hour at room temperature. The primary antibody against AD5 (Cat. No. ab6982, Abcam) diluted with PBS containing 1% BSA was used and incubated with the cells overnight at 4°C. The next day, the cells then were washed and incubated with fluorochrome-conjugated secondary antibodies for 2 hours at room temperature. The cells then were washed and mounted with antifade mounting media containing DAPI. The image acquisition and assembly were done with an ImageXpress Pico scanner from Molecular Devices. The images were taken to assess the binding and trafficking of HPK at different times. The image analysis was carried out with ImageJ software.

## Statistical analysis

**Figure 1:**
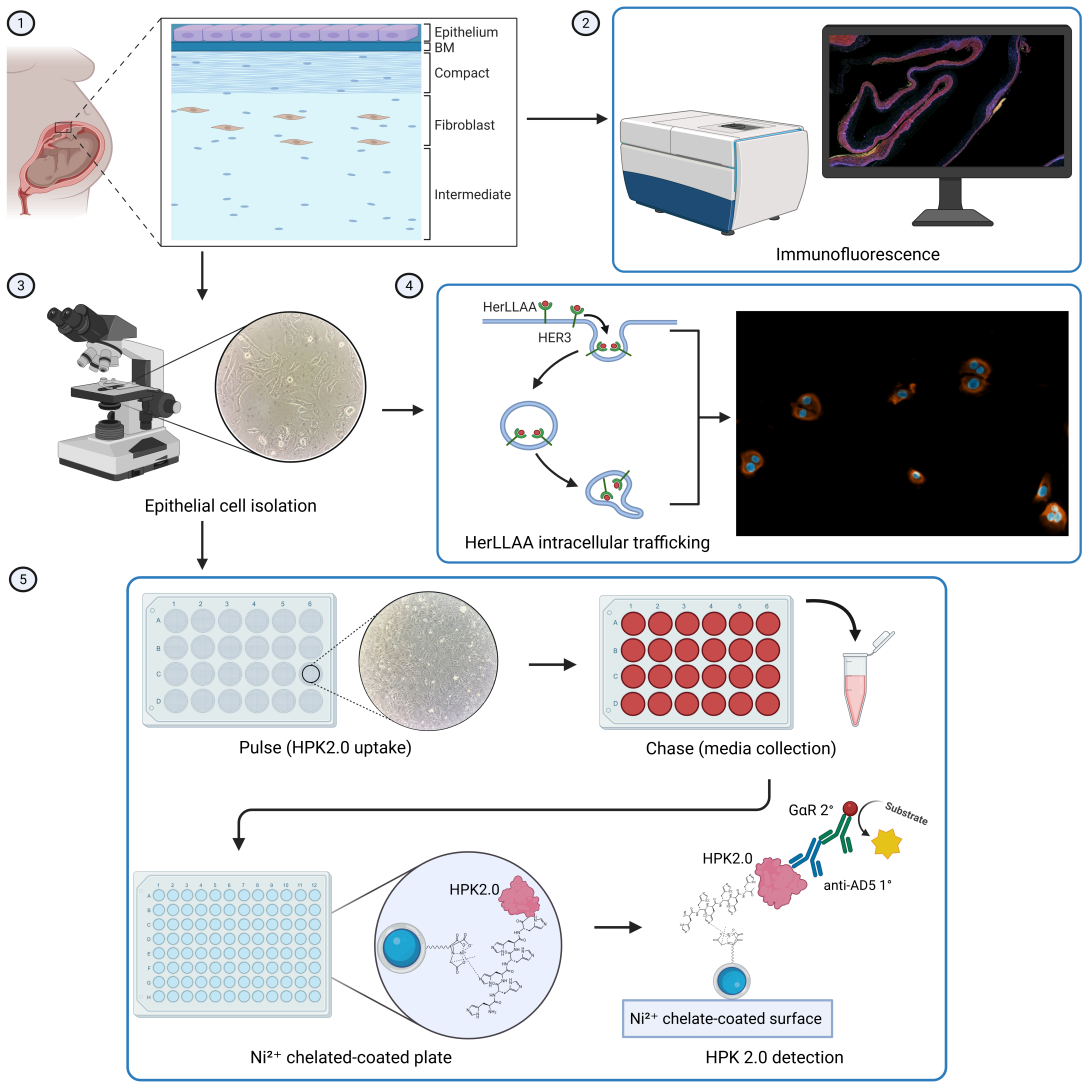
Experimental workflow for HER3 characterization and HPK2.0 uptake-release analysis in human amniotic epithelial cells (hAECs). (1) A schematic representation of placental compartmentalization is shown, including epithelial layer, basement membrane (BM), compact, fibroblast, and intermediate layers of the amnion, highlighting the regions used for downstream analysis. (2) Immunofluorescent imaging of processed FFPE tissues showing pan-cytokeratin and HER3 detection in placental sections. (3) Isolation of epithelial cells from amniotic membranes followed by *in vitro* culture expansion prior to functional assays. (4) Schematic depiction of HER3-mediated intracellular trafficking of HerLLAA following receptor engagement, internalization, and subcellular transport. (5) Pulse-chase assay design for quantitative analysis of HPK2.0 uptake and release, including nickel (Ni^2+^) capture of His-tagged HPK2.0 and detection using anti-AD5 primary antibody and secondary antibody with substrate development. This workflow enables quantification of intracellular retention and extracellular release dynamics. Created in BioRender. Aceves, J. (2026) https://BioRender.com/pu1ui0z

Statistical analyses were performed using GraphPad Prism version 10.6.1 (GraphPad Software, Inc., San Diego, CA, USA). Data are presented as mean ± standard deviation (SD). Comparisons were conducted using Student’s t-test or two-way ANOVA, as appropriate. A p value < 0.05 was considered statistically significant. A schematic of the overall study workflow is shown in Fig. 1.

## Results

### Amniotic Membrane, Umbilical Cord and hAECs Express High HER3 Levels

**Figure 2:**
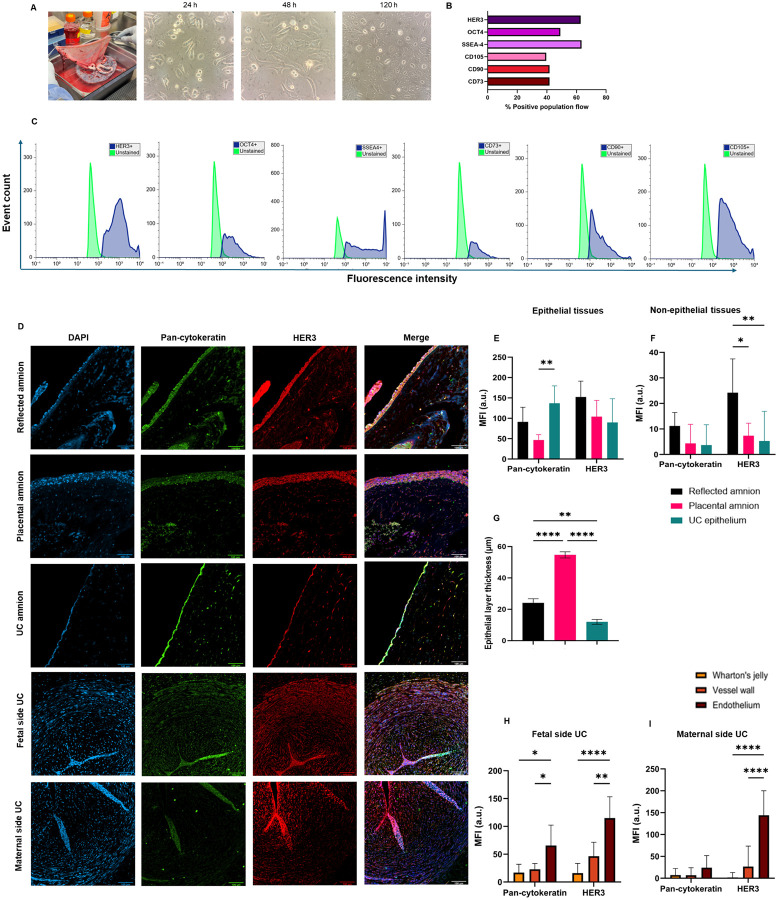
HER3 expression profiling in placental compartments and phenotypic characterization of isolated hAECs. **(A)** Representative gross placental handling and brightfield images of isolated epithelial cells at 24 hr, 48 hr, and 120 hr post-isolation, demonstrating epithelial morphology and adherence. **(B)** Flow cytometric quantification of HER3, OCT4, SSEA4, CD105, CD90, and CD73, presented as percentage of positive population. **(C)** Representative fluorescence histograms comparing stained versus unstained controls for HER3, OCT4, SSEA4, CD105, CD90, and CD73. **(D)** Immunofluorescence staining of reflected amnion, placental amnion, umbilical cord amnion, and fetal and maternal side umbilical cord regions, showing DAPI (nuclei), pan-cytokeratin (epithelial marker), HER3, and merged images. **(E–F)** Quantification of mean fluorescence intensity (MFI) for pan-cytokeratin and HER3 across epithelial layer (E) and other amniotic compartments (F). **(G)** Measurement of epithelial layer thickness across reflected, placental, and umbilical amnion, demonstrating significant regional variation. **(H–I)** Quantitative analysis of pan-cytokeratin and HER3 in Wharton’s jelly, vessel wall, and endothelial compartments of fetal (H) and maternal (I) side umbilical cord, demonstrating significant compartment-specific enrichment. Data are presented as mean ± SD. Statistical significance is indicated as *p < 0.05, **p < 0.01, and ****p < 0.0001, as shown in the respective panels.

Freshly isolated hAECs adhered readily to tissue culture plastic and maintained a cobblestone epithelial morphology for up to 120 hours in culture, indicating sustained viability and adherence (Fig. 2. A).

We evaluated the expression of stemness and mesenchymal related markers as well as HER3 in hAECs. Flow cytometry analysis demonstrated that hAECs expressed low to moderate levels of mesenchymal-associated markers CD73 (41.84%), CD105 (39.58%), and CD90 (41.84%). Stemness-associated markers SSEA4 (63.39%) and OCT4 (49.10%) as well as HER3 (62.78%) were also detected. The results indicated heterogeneous yet consistent marker expression, with the highest positive fractions observed for HER3, OCT4 and SSEA-4, followed by CD73, CD105, and CD90 (Fig. 2. B–C).

Immunofluorescence analysis was performed to evaluate the distribution of pan-cytokeratin and HER3 across amniotic samples of various regions (Supplementary Fig. S1) and umbilical cord (UC) compartments (Supplementary Fig. S2). The results showed well-defined epithelial architecture in reflected amnion, placental amnion, and UC epithelium, with strong pan-cytokeratin staining defining the epithelial layer (Fig. 2. D). HER3 immunoreactivity was detected in amniotic samples from all regions, predominantly localized along the epithelial layer, with variable intensity across compartments situated directly underneath the epithelial layer. Merged images confirmed co-localization of HER3 within cytokeratin-positive epithelial cells. Deeper stromal regions showed negligible epithelial marker expression but retained some compartment-specific HER3 signals. Quantitative analysis of fluorescence intensity within the epithelial layer of samples from reflected amnion, placental amnion, and UC epithelium revealed significantly higher pan-cytokeratin expression in UC epithelium compared to placental amnion (p < 0.01) (Fig. 2. E). In deeper stromal regions HER3 expression was higher in reflected amnion samples compared to placental and UC epithelium (p < 0.05; p < 0.01) (Fig. 2. F). Measurement of epithelial layer thickness demonstrated marked regional variation. The placental amnion exhibited significantly greater epithelial thickness compared to reflected and UC epithelium (p < 0.0001), while reflected amnion was also significantly thicker than UC epithelium (p < 0.01) (Fig. 2. G).

Further compartmental analysis of fetal and maternal side UC regions revealed distinct distribution patterns. In the fetal side, HER3 expression was significantly enriched in the endothelial compartment compared to vessel wall and Wharton’s jelly (p < 0.01; p < 0.0001). Although pan-cytokeratin remained low outside epithelial structures, endothelial expression in the fetal-side UC was higher than in the vessel wall and Wharton’s jelly (p < 0.05) (Fig. 2. H). Similarly, in the maternal side UC, HER3 intensity was markedly elevated in endothelial regions compared to surrounding stromal components (p < 0.0001), demonstrating compartment-specific enrichment (Fig. 2. I). Together, these findings demonstrate that HER3 exhibits region-specific distribution and underscore its relevance as both a potential off-target and therapeutic target in placental-derived cells.

### hAECs Release Stable HER3^+^ Exosomes Over Time

**Figure 3:**
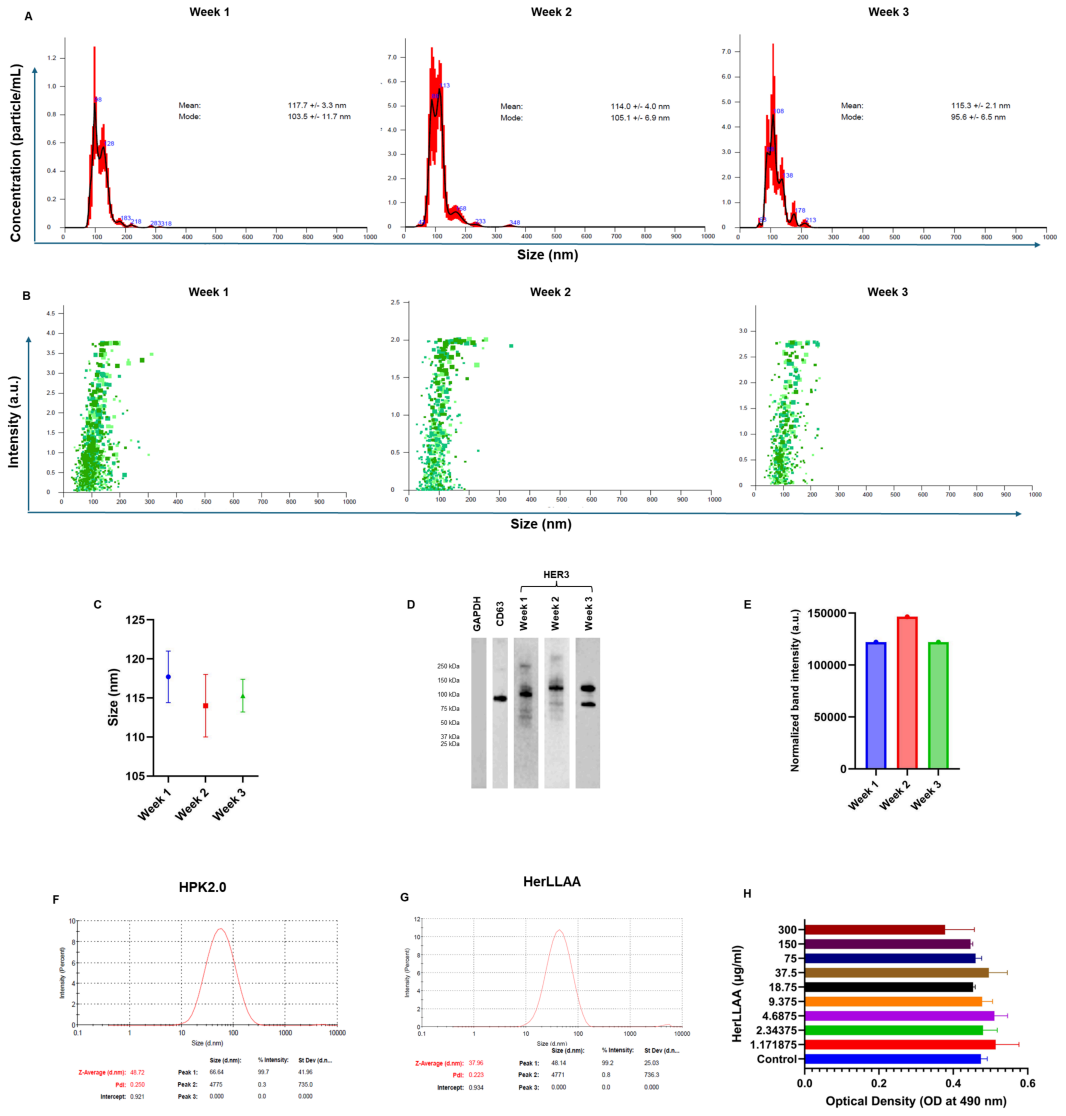
Characterization of hAEC-derived exosomes and HPK2.0 particles. **(A)**Nanoparticle tracking analysis (NTA) showing particle concentration (particles/mL) versus size (nm) for Week 1, Week 2, and Week 3 isolations. **(B)**Scatter plot of particle intensity vs. size with calculated mean and mode diameters to show consistent vesicle populations centered around 100–120 nm. **(C)**Quantitative comparison of the mean vesicle sizes for each week to ensure reproducibility (p > 0.05). **(D)** Western blotting of exosome lysate to detect CD63, the exosome marker; ErbB3/HER3; and GAPDH, the control. **(E)**Densitometric quantification of normalized band intensity for ErbB3 / HER3 across weeks. **(F–G)** Representative distribution curves showing peak sizes and intensity profiles for HPK2.0 (F) and HerLLAA (G). **(H)** Metabolic assay of HerLLAA for hAECs showed no toxicity across concentrations compared to control group (p > 0.05). Data are presented as mean ± SD.

Exosomes isolated from hAEC-conditioned medium exhibited stable and consistent size distributions across sequential time points, with nanoparticle tracking analysis (NTA) confirming that a predominant population matching the expected exosome size range exhibited mean particle diameters of 117.7 ± 3.3 nm (Week 1), 114.0 ± 4.0 nm (Week 2), and 115.3 ± 2.1 nm (Week 3) (Fig. 3. A). Scatter plots of particle size versus intensity confirmed that the vesicle population sustained longitudinal homogeneity with no detectable shift toward larger aggregates (Fig. 3. B). Quantitative comparison confirmed no significant variation in average particle size between isolation batches (p = 0.73) (Fig. 3. C).

Exosome identity was validated by immunoblotting which confirmed that the exosome-specific marker CD63 was present in exosome lysates (Fig. 3. D). HER3 also tracked with these lysates which exhibited sustained HER3 signal intensity longitudinally and between isolations (Fig. 3. E). These findings indicate that HER3 is present on hAEC-derived exosomes which in turn can be reproducibly isolated with sustained physical properties, suggesting that these exosomes may serve a role in HER3-associated placental transport or signaling processes.

### HER3-Targeted HPK and HerLLAA Exhibit Stable Nanostructures and are Nontoxic to hAECs

Membrane-impermeable therapeutics must overcome tumor-associated biological barriers to access intracellular targets, creating a need for delivery systems that combine efficient membrane penetration with molecular specificity. To achieve HER3-directed targeting, HPK was engineered using the receptor-binding domain of the HER3 ligand neuregulin-1α as its targeting module. This domain confers selective binding to HER3, a receptor frequently overexpressed in aggressive and therapy-resistant tumors^11,12,16^. Structurally, HPK is built on the adenoviral penton base protein, a self-assembled homopentameric ring that caps each vertex of the icosahedral adenovirus capsid and mediates membrane penetration. In HPK, the neuregulin-1α receptor-binding region is produced as an N-terminal fusion to the penton base, enabling receptor-specific engagement of HER3-positive cells. A C-terminal decalysine tail provides electrostatic interaction with anionic cargo, allowing encapsulation and delivery following HER3-mediated binding and internalization. This integrated design couples HER3-selective targeting with membrane penetration and cargo encapsulation to support efficient intracellular delivery^26^ (Supplementary Fig. S3). Supplementary Video S1 and S2 show the HPK2.0 and HerLLAA molecular dynamics in 3D models. In the present study, we use a second generation version, HPK2.0, which has acquired improvements in: biomanufacturing yield, purity and homogeneity; cargo loading; receptor specificity; and therapeutic delivery and efficacy.^27^

To evaluate the potential of HPK2.0 as a HER3-targeting agent on hAECs, we assembled purified HPK2.0 -- which isolates as a monodisperse population (Fig. 3. F) -- with the inert oligonucleotide cargo designated LLAA to form the nanobioparticle HerLLAA, which also forms a largely uniform monodisperse particle population with sustained colloidal stability (Fig. 3. G).

The impact of HerLLAA on hAEC viability was evaluated across a wide concentration range (1.17–300 μg/mL). As the LLAA cargo lacks any functional activity, HerLLAA serves as an empty particle whose impact on cell viability would reflect any potential toxicity contributed by the assembled HPK shell. All concentrations lacked any significant reduction in metabolic activity or cell viability (p > 0.05), indicating that HerLLAA is safe on hAECs over a broad concentration range (Fig. 3. H).

### hAECs Efficiently Internalize HPK2.0 and Release It in a Dose- and Time-Dependent Manner

**Figure 4:**
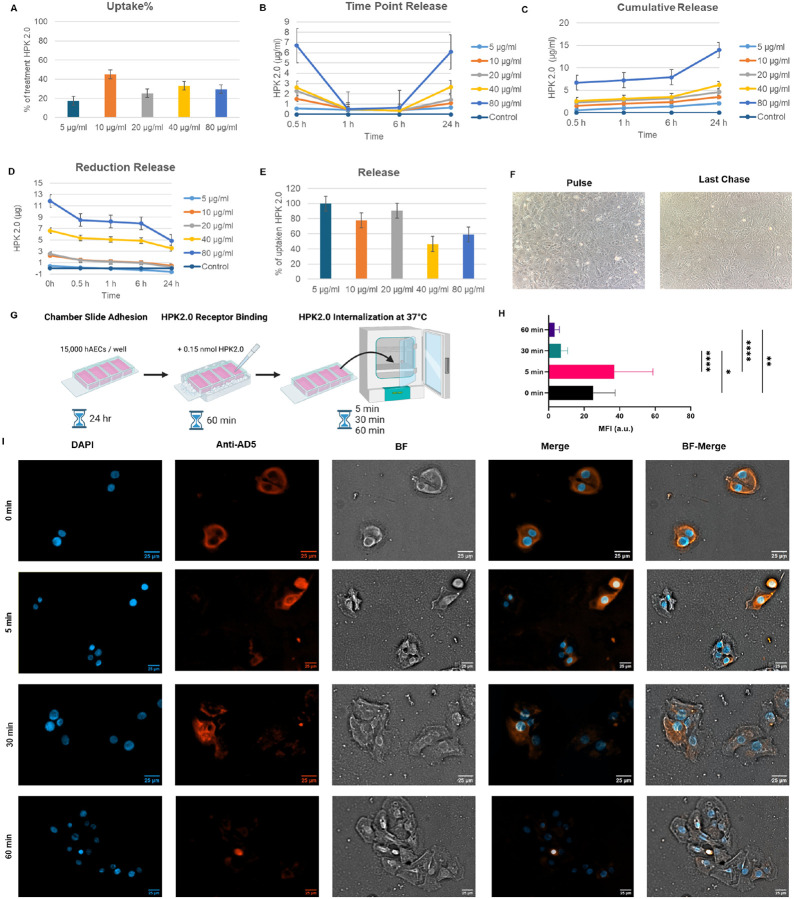
Dose-dependent uptake, intracellular trafficking, and release kinetics of HPK2.0 in hAECs. **(A)**Percentage uptake of HPK2.0 relative to administered dose (5–80 μg/mL), demonstrating a non-linear uptake pattern across concentrations. **(B)** Time-point release of HPK2.0 (0.5 hr, 1 hr, 6 hr, 24 hr) expressed as μg/mL of HPK2.0 in the conditioned media. **(C)** Cumulative release curves over time for each treatment concentration. **(D)** Reduction in intracellularly retained HPK2.0 over time, showing increasing secretion. **(E)** Percentage of released HPK2.0 compared to uptaken HPK2.0. **(F)** Representative brightfield images comparing pulse (granulated) and final chase (clear) hAECs morphology. **(G)** Schematic of trafficking assay including receptor binding on ice and HPK2.0 internalization at 37°C at specified time points. **(H)**Quantification of intracellular fluorescence intensity (MFI) at 0, 5, 30, and 60 minutes showing significant reduction over time. **(I)** Immunofluorescence images (DAPI, anti-AD5, brightfield, merged) demonstrating transition from membrane signal at early time points to intracellular punctate localization and finally release at later time points. Data are presented as mean ± SD. Statistical significance is indicated as *p < 0.05, **p < 0.01, and ****p < 0.0001, as shown in the respective panels. Created in BioRender. Aceves, J. (2026) https://BioRender.com/pu1ui0z

Vesicular transcytosis across the amnion can occur through the capacity of hAECs to rapidly internalize and release macromolecules, including chemotherapeutic agents, in a time-dependent manner.^23,24^. Accordingly, we evaluated HPK2.0 uptake and subsequent release by hAECs using a pulse-chase assay across a range of input concentrations (5–80 μg/mL). Following a 1 h pulse, HPK2.0 uptake increased with higher input concentrations resulting in greater intracellular accumulation at the end of the uptake phase. Calculation of intracellular HPK2.0 after 1h treatment showed a non-linear, bell-shaped distribution, with reduced uptake at both low and high concentrations and peak retention at the intermediate dose (10 μg/mL) (Fig. 4. A). During the release (chase) phase, HPK2.0 was detected in conditioned media as early as 0.5 h after medium replacement. Time-point analysis revealed an initial burst release followed by reduced levels at 1 h and 6 h, and a secondary increase at 24 h (Fig. 4. B). Cumulative release analysis showed a time-dependent increase in total HPK recovered in the supernatant, with the highest cumulative release observed at 24 h for cells pulsed with 80 μg/mL HPK2.0 (Fig. 4. C). These findings corresponded with a progressive decrease in intracellular HPK2.0 levels during the chase period, consistent with depletion of intracellular HPK2.0 pools (Fig. 4. D). Release efficiency varied by dose and was not linear. The lowest release fraction was observed at 40 μg/mL, whereas higher release fractions were observed at 5 and 20μg/mL, with intermediate values at 10 and 80 μg/mL (Fig. 4. E). The hAECs exhibited highly granulated morphology after treatment with HPK2.0 and recovery of normal morphology after the last chase timepoint (Fig. 4. F), indicative of transitory macromolecular uptake. These findings strongly suggest that hAECs not only efficiently internalize HPK2.0 but also actively release it into the extracellular environment in a dose- and time-dependent manner, supporting a dynamic transcellular handling of HER3-targeting agents.

### HerLLAA Binding to hAECs Is Followed by Rapid Internalization and Post-Entry Re-Distribution

To examine how the early stages of HPK2.0 cell entry and release comports with the binding and intracellular trafficking of HerLLAA, we interrogated HerLLAA-treated hAECs using a time-resolved *in vitro* pulse-chase assay (Fig. 4. G) using immunofluorescence detection of HerLLAA to track intracellular distribution. Measurements of overall intracellular fluorescence intensity indicate that the highest fluorescence quantum yield of HPK2.0 peaks at 5 min after initial cell binding followed by reduced intensities at 30 min and 60 min (p < 0.0001 compared to the 5 min time point) (Fig. 4. H). The spatial distribution of HerLLAA at these time points agree with a predominantly cell surface localization early after cell treatment (0 min) followed by pronounced intracellular accumulation and widespread cytoplasmic distribution at 5 min that becomes more diffuse by 30 min and 60 min after HerLLAA uptake (Fig. 4. I). These results suggest that nanobioparticle binding to hAECs is rapidly followed by internalization and dynamic intracellular trafficking, culminating in time-dependent depletion of intracellular nanobioparticles, which -- taken together with the earlier uptake and release dynamics -- comport with active release of internalized nanobioparticles rather than long-term sequestration.

## Discussion

In this study, we investigated the interaction of HER3-targeted nanotherapeutics with hAECs, a placental epithelial cell type in direct contact with the fetus and amniotic fluid. We found that hAECs express HER3, including on their secreted extracellular vesicles, and efficiently internalize HER3-targeting nanobioparticles, subsequently releasing them in a dose- and time-dependent manner without detectable cytotoxicity. These findings indicate that hAECs actively engage with HER3-directed agents rather than serving as passive barriers, with implications for placental exposure during pregnancy and potential use of HPK bioparticles as a platform for targeted therapeutic delivery to the placenta and fetus.

hAECs showed a heterogeneous phenotypic profile characterized by expression of stemness- and mesenchymal-associated markers, consistent with prior reports describing their transitional epithelial characteristics^28–30^. Considering this profile, we identified substantial HER3 expression in isolated hAECs, extending the biological context of this receptor beyond malignant tissues. While HER3 has been described in trophoblast populations, its expression in amniotic epithelial cells and placenta-derived extracellular vesicles has received limited attention^31,32^. The correlation between cellular and tissue-level analyses suggests that the AM and UC represent normal HER3-positive compartments at the maternofetal borders. However, our analysis was restricted to term placentas, and the functional role of HER3 in placental epithelial biology was not directly assessed during earlier gestation, when placental architecture and transport dynamics are distinct. More studies examining gestational variability, pathological contexts, and downstream signaling activity may define the physiological and pathological relevance of amniotic HER3 expression.

The AM is increasingly recognized as a dynamic organ that regulates molecular exchange between the maternal and fetal compartments^33^. hAECs are known to participate in vesicular transport and transcytosis of macromolecules^23^. Consistent with this role, the current study suggests that hAECs actively transcytose HER3-targeted nanobioparticles rather than passively retaining them. This agrees with our recent studies demonstrating that these nanobioparticles traverse the blood-brain barrier using a HER3-mediated transcytosis pathway supported by caveolae trafficking^11^. The non-linear uptake profile in hAECs suggested regulated trafficking mechanisms such as receptor saturation, recycling, or vesicular trafficking^34^. Similar transport behaviors have been described for other receptor-mediated ligands in the placenta, including IgG and iron, where intracellular accumulation is tightly controlled^35–37^. However, these dynamics were observed in a simplified *in vitro* system lacking maternal circulation, trophoblast layers, and immune components that normally shape placental transport *in vivo*. Therefore, while our findings suggest that placental exposure to HER3-targeted agents may be biologically regulated rather than purely passive interactions, *in vivo* pharmacokinetic studies may help determine whether maternal dosing directly translates to fetal exposure.

Controlled transcellular passage is fundamental for placental drug delivery aimed at achieving maximum therapeutic exposure while minimizing off-target toxicity in placental and fetal pathologies, including preeclampsia and eclampsia^34,38,39^. In this context, HPK2.0’s design makes it a promising platform for delivering small molecules, nucleic acids, or biologics to the placenta or fetus^40,41^. *In vivo* validation– including direct trans-placental passage, fetal biodistribution, and systemic pharmacokinetics, have yet to be evaluated before clinical translation can be considered. Nevertheless, the presence of HER3 on hAECs and its connection with transcytosis of HER3-directed nanobioparticles highlights a potential new route of transport across this placental barrier while demonstrating that the HPK technology may be worth considering as a platform to develop for placenta-directed therapeutic strategies.

## Conclusion

In conclusion, placental tissues and hAECs express HER3 and actively engage with HER3-targeted nanotherapeutics through regulated uptake, intracellular trafficking and release, without evidence of cytotoxicity. These findings identify the placenta as an active biological interface for targeted agents and highlight HPK bioparticles as a viable platform for delivering therapeutic cargo to the placenta and fetus.

## Supplementary Material

Supplementary Files

This is a list of supplementary files associated with this preprint. Click to download.

- 1PublicationLicenseMar092026.pdf
- 2PublicationLicenseMar092026.pdf
- S1.png
- S2.png
- S3.png
- SuppVideo1.mp4
- SuppVideo2.mp4
- supplementaryFigureLegend.docx

## Data Availability

The datasets generated and analyzed during this study, including whole-slide immunofluorescence images of placental tissues, are available in the Zenodo repository (10.5281/zenodo.18943037). Additional data supporting the findings are included within the article and its supplementary materials.
